# Combined Exposure to Endocrine Disruptors, BPA and BP-3, during Pregnancy and Lactation Alters Postnatal Body Mass, Growth, White Adipose Tissue Morphology, and Adipogenic Gene Expression in a Sex-Specific Manner

**DOI:** 10.1021/envhealth.5c00679

**Published:** 2026-04-20

**Authors:** Kristína Raticová, Julia Howanski, Beate Fink, Stefan Röder, Mario Bauer, Anne Schumacher, Ana C. Zenclussen, Tobias Kretschmer

**Affiliations:** † Department of Environmental Immunology, 28342 Helmholtz Centre for Environmental Research - UFZ, Leipzig 04318, Germany; ‡ Perinatal Immunology, Saxonian Incubator for Clinical Translation (SIKT), Medical Faculty, Leipzig University, Leipzig 04103, Germany; § Leipzig Reproductive Health Research Center (LE-REP), Leipzig University, Leipzig 04103, Germany; ∥ German Center for Child and Adolescent Health (DZKJ), Partner Site Leipzig/Dresden 04103, Germany; ⊥ LeiCeM - Leipzig Center of Metabolism, Leipzig University, Leipzig 04103, Germany

**Keywords:** endocrine-disrupting chemicals (EDCs), bisphenol A (BPA), benzophenone-3 (BP-3), adipogenesis, white adipose tissue (WAT), metabolic programming, PPARγ, FABP4, metabolic disorders

## Abstract

Endocrine-disrupting chemicals (EDCs) like bisphenol A (BPA) and benzophenone-3 (BP-3) are omnipresent and previously linked to various metabolic disorders. As EDC exposure already begins during prenatal development, we investigated the effects of BPA and BP-3 exposure during gestation and lactation using a murine model on adipogenesis and metabolic pathways in white adipose tissue (WAT) in the offspring. We monitored postnatal body mass and analyzed the morphology and gene expression of WAT in adult male and female offspring. BPA- and BPA+BP-3-exposed males showed an increase in body mass in early adulthood, whereas BP-3-exposed females presented a decreased growth rate later in life. We identified altered adipocyte area and distribution, suggesting hyperplasia in the WAT of BPA and BPA+BP-3-exposed males. Gene expression analysis showed Pparg upregulation in BPA+BP-3-exposed males and Fabp4 and Adipoq downregulation in BP-3-exposed females. Sex-specific associations between gene expression, adipocyte area, and body mass in controls were disrupted by EDC exposure. Our findings suggest increased postnatal body mass and Pparg-mediated WAT hyperplasia in BPA+BP-3-exposed male mice. Fabp4 and Adipoq downregulation may be responsible for counteracting the BP-3-induced metabolic dysregulation in females. This calls for consideration of sex-specific differences of endocrine disruption in the future development of preventive and therapeutic targets for metabolic disorders.

## Graphical abstract

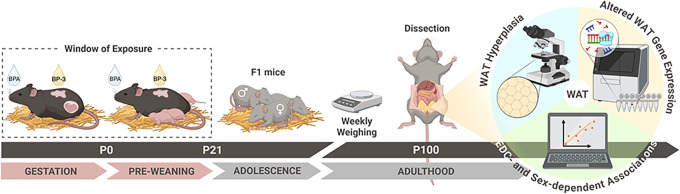

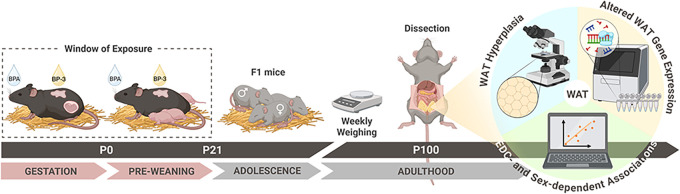

## 1 Introduction

In recent years, the potential hormonal activity of various chemicals has become a major topic of scientific research, resulting in public attention. Consequently, many plastic and personal care products in particular are being marketed as free of endocrine-disrupting chemicals (EDCs). However, only substances with confirmed endocrine-disrupting potential are being avoided, while many new chemicals remain untested. Exposure to EDCs can take place via ingestion, inhalation, dermal, subcutaneous, and intravenous application, e.g., by contact to medical equipment. Several studies confirmed the presence of EDCs in human and neonatal blood, urine, placenta, and amniotic fluid.^1–5^ First links between chemical exposure and reproductive as well as developmental abnormalities were reported in the 1970s.^6^ The following decades of research uncovered the ability of EDCs to bind hormonal receptors, interfere with physiological endocrine regulation, and thereby lead to increasing rates of endocrine-related metabolic diseases, many of which are associated with obesity.^7–9^

According to the WHO, 2.5 billion adults (18 years and older) and 390 million children and adolescents aged 5–19 years were overweight in 2022. Since 1990, the prevalence of obesity has more than doubled in adults and quadrupled in adolescents.^10^ Besides a number of related health complications, severely obese children suffer from a diminished quality of life comparable to that of children with chronic diseases such as cancer.^11^ Moreover, an analysis of the Global Burden of Disease Study 2021 revealed a major rise in obesity-related deaths, from 1.46 million in 1990 to 3.79 million in 2021. These numbers translate to an increase of nearly 154%.^12^

White adipose tissue (WAT), as the major storage tissue for excess calories and fats, is one of the body’s most commonly overlooked endocrine organs. White adipocytes function as energy storage, with their metabolic pathways being tightly regulated by hormones like insulin, leptin, glucocorticoids, estrogens, and thyroid hormones. Additionally, WAT secretes adipokines, a distinct group of signaling molecules that mediate lipid and glucose metabolism, tissue inflammation, insulin sensitivity, appetite, and immune responses.^13^ Disruption of these closed-loop regulatory systems can lead to metabolic disorders, insulin resistance, and type 2 diabetes mellitus (T2D), dysregulated lipid metabolism, and obesity. The complex disease pattern is no longer seen as “just” a combination of excessive caloric intake and reduced energy expenditure. After the first associations between the increasing prevalence of obesity and chemical burden on the environment were published, Grün and Blumberg introduced the obesogen hypothesis in 2006.^14^ The term “obesogen” underwent multiple changes in meaning and is nowadays defined as a “xenobiotic chemical that can disrupt the normal development and homeostatic control of adipogenesis and/or energy balance”.^15^ Thus, obesogens contribute to obesity either by increasing the number of adipocytes or by increasing lipid accumulation in existing adipocytes. Obesogens might additionally promote obesity by altering the body’s natural metabolic functioning or hormonal regulation of hunger and satiety.^16–19^ As a result, although not being biologically needed, a shift of metabolism toward calorie storage occurs. In 2015, it was proposed to change the term “obesogen hypothesis” to “metabolism-disrupting chemical hypothesis” due to new evidence of EDCs acting to increase the susceptibility for T2D and metabolic syndrome.^20^ Due to differences in male and female endocrine systems, sex-specific differences in the impact of EDCs and the progress of these diseases are to be expected.

Two of the most prevalent EDCs in everyday life are bisphenol A (BPA) and benzophenone-3 (BP-3). BPA is a common ingredient in plastics and resins with the purpose to improve the durability, transparency, and heat resistance of plastic products. Besides being banned in cosmetics, thermal paper and baby bottles in the European Union, the European Food Safety Authority (EFSA) has set a tolerable daily intake (TDI) for BPA at 4 μg per kilogram of bodyweight per day (μg/kg/d) in 2015.^21^ In 2023, the TDI was further decreased by a factor of 20,000 to 0.2 ng/kg/d.^22^ BPA urinary prevalence was measured in the 2013–2014 U.S.-based National Health and Nutrition Examination Survey (NHANES) and was found in 95% of urine samples of the study population.^23^ In Europe, similar BPA prevalence in human populations were reported for women from 2007–2014 in the European Joint Programme HBM4 EU.^24^ BPA has been reported to be associated with obesity, insulin resistance, and T2D in humans and with metabolic diseases in animals as well.^25^ Besides BPA, another chemical that has recently gained attention as a potential EDC is BP-3. BP-3 is used as a UV filter in commercially available sunscreens and other cosmetics. In 2021, the Scientific Committee on Consumer Safety (SCCS) published a report on BP-3 not being safe for consumers at a concentration of 6% as sunscreen protection in body creams due to its endocrine-disrupting properties. A reduction of the concentration to a maximum of 2.2% BP-3 in body creams was recommended.^26^ BP-3 has been found in urine samples of 66–98% of the human population in Europe^27^ and up to 96.8% in the US population.^28^ Moreover, BP-3 has been associated with increased birth weight and higher odds of obesity in humans.^25,29^ So far, only limited research on the effects of gestational and lactational BP-3 exposure has been published.

There is evidence that environmental disruption during gestation and neonatal development can lead to increased susceptibility to disease later in life through subtle changes in gene expression, DNA methylation, histone modification, and disruption in developmental programming.^30^ This suggests that exposure to critical doses of EDCs during vulnerable windows in development may lay the foundation for the development of metabolic diseases such as insulin resistance, T2D, and obesity, especially when additional predisposing factors for these multifactorial conditions are present. Real-life exposure to EDCs never occurs to exclusively one chemical at a time but rather to a mixture of chemicals over prolonged periods during life. Hence, the evaluation of cumulative or combined effects of these chemicals is essential in order to assess their true potential in disrupting endocrine and metabolic functions. To date, no studies have specifically examined the effects of combined gestational and lactational exposure to BPA and BP-3 on metabolic outcomes. Despite the fact that both chemicals are recognized as EDCs and are prevalent in human exposure, their potential additive, synergistic, or antagonistic interactions with regard to metabolic disruption remain undetermined.

In this study, we aimed to investigate sex-specific effects of a combined gestational and lactational exposure to BPA and BP-3 at concentrations relevant to human exposure on offspring body mass, growth, and WAT development in male and female mice. By analyzing the transgenerational impact of such an exposure to these EDCs on WAT and associated metabolic pathways, we intended to identify potential endocrine-disrupting mechanisms in WAT that may contribute to the development of obesity and related metabolic disorders.

## 2 Materials and Methods

### 2.1 Animal Housing

All procedures of animal housing, treatment, and experiments for this study were conducted after approval by the German local authorities (Animal protection commission of the Landesdirektion Sachsen, Germany, case number: TVV21/21). Only authorized individuals were involved in animal handling, treatment, and experiments according to the Guide for Care and Use of Animals in Agriculture Research and Teaching. C57BL/6 female and BALB/c male mice at the age of 8 weeks were purchased from Janvier Laboratories (Le Genest-Saint-Isle, France) and housed at the animal facility of the Saxonian Incubator for Clinical Translation, University of Leipzig. During the entirety of experimental conduct, including an acclimatization period of at least 7 days, the animals were kept at 22 ± 1 °C on a 12 h (6 am/6 pm) light/dark cycle under specific pathogen-free (SPF) conditions, allowing ad libitum access to water and a standard chow diet (#V1534-300, sniff). From gestation day (GD) 12 onward, the female mice were provided with breeding chow (#V1185-300, sniff) ad libitum. To achieve an allogeneic pregnancy, which translates better to a “natural” pregnancy with immunogenic and genetic heterozygosity compared to an autogenic pregnancy of an inbred strain, BALB/c males and C57BL/6 females were paired in a one-to-one ratio. The female mice were monitored for the presence of a vaginal plug every morning and late afternoon. The day of plug detection was considered to be GD0 and the pregnant dams were randomly assigned to the following 4 treatment groups: vehicle (*n* = 11), BPA (*n* = 11), BP-3 (*n* = 14), and BPA+BP-3 (*n* = 13) [24]. Thus, only pregnant C57BL/6 females were exposed to EDCs to reproduce exposure during pregnancy and lactation. The number of animals used was based on a statistical power calculation for the approval of the animal experiment by the German local authorities (case number: TVV21/21). Animals were kept in groups of 3–4 until GD16 and separated into individual cages from GD16 onward, to limit disturbances during parturition and until the end of lactation on postnatal day 21 (P21). After weaning, F1 generation, BALB/c-C57BL/6 offspring were housed in individual cages with same-sex groups of 2 to 5 animals and provided with a standard chow diet.

### 2.2 Treatment of Dams

Pregnant dams were treated with a vehicle substance, a single EDC (BPA or BP-3) or a mixture of 2 common EDCs (BPA+BP-3 coexposure) from GD0 until the end of lactation on P21.^31^ Briefly, stock solutions of BPA (#239658, Sigma-Aldrich) of 400 μg/L in 0.01 vol % ethanol and BP-3 (#H36206, Sigma-Aldrich) of 10 mg/mL dissolved in commercial olive oil (Ja!, REWE Group) were prepared. To the BPA and BPA+BP-3 treatment groups, BPA was administered via oral gavage using a flexible feeding tube (Instech Laboratories) at 10 μL/g/d, which translates to 4 μg/kg/d. This equals the tolerable daily intake (TDI) of BPA that was set by the European Food Safety Authority (EFSA) in 2015 (European Food Safety Authority, 2015). BP-3 was applied dermally at 5 μL/g/d to a 2 × 2 cm shaved spot on the upper back of the dams in the BP-3 and BPA+BP-3 treatment group. To ensure a total absorption of the solution, they were separated into an empty cage for 5 to 10 min after application in order to prevent grooming by other animals. The treatment results in an exposure to BP-3 at 50 mg/kg/d, which is equivalent to a single application of 2 mg/cm^2^ of products containing 10% (w/w) BP-3 on the skin of the whole body.^32^ The human BP-3 exposure dose was previously translated to our mouse model, with BP-3 being detected in relevant doses in maternal serum and amniotic fluid of dermally exposed dams. Animals of the respective vehicle groups were treated with commercial olive oil (5 μL/g/d) and 0.01% ethanol (10 μL/g/d) using the same application methods. After birth, all offspring of each litter were weighed and sexed according to the anogenital distance on P1, P7, P14, and P21. From P21 onward, the body mass of all male and female animals from each litter was determined weekly. After weaning on P21, each offspring was tagged to allow individual documentation of body mass until euthanasia. For each experiment, 1 to 2 male or female pups per litter were used.

### 2.3 Histological Tissue Processing of WAT

On P100, male and female offspring from each of the four exposure groups were euthanized by cervical dislocation. Blood and tissue samples from the epigonadal WAT were collected. Only the WAT was used for histological analysis. Following dissection, the tissue was fixed in 10% natural-buffered formalin (Histofix, Carl Roth) for a maximum of 24 h and dehydrated in ethanol solutions of increasing concentration and acetic acid *n*-butylester before being embedded in paraffin. A total number of 5 tissue slices per WAT sample of each animal were cut using a rotary microtome, cutting at a thickness of 5 μm and leaving a minimal distance of 200 μm between each section to avoid including the same set of adipocytes in more than one slice. Afterward, the sections were transferred onto polylysine-coated slides (Superfrost) and air-dried at 40 °C overnight. Resulting sections were dewaxed in Histoclear (Carl Roth) and rehydrated before staining with hematoxylin and eosin (H/E, Carl Roth) following a standard protocol. Images of 5 randomly selected sites of each section were captured using the Keyence BX800 digital microscope at 40-fold magnification.

### 2.4 Analysis of Adipocyte Area

All measurements of adipocytes were obtained with the aid of the Java-based image processing software “ImageJ” v13.0.6. The investigator was blinded to the experimental group of the sample. The area of each adipocyte was determined using the “Analyze Particle” function after converting its grayscale (8-bit) image to a binary mask via thresholding the cell membranes from the unilocular lipid droplet in the cytoplasm. Cells bordering the edges of the image were excluded from the analysis, as well as particles with an area less than 50 μm^2^. Adipocytes with perforated cell membranes were separated manually using the “Drawing Tool”.

### 2.5 Quantitative Real-time PCR (RT-PCR)

WAT tissue was snap frozen in liquid nitrogen after dissection and stored at −80 °C. After thawing, 500 μL of Trizol Reagent (Invitrogen, Carlsbad, California, USA) was added to each sample prior to homogenization in a tissue lyser (4 min, 50 1/s, 1 stainless steel bead (Qiagen) in each vial). Total RNA isolation was performed according to the manufacturer’s instructions using peqGold PhaseTrap tubes (peqlab, Erlangen, Germany). The RNA concentration was measured via a NanoDrop spectrophotometer (Thermo Fisher Scientific) and cDNA was synthesized from 1000 ng RNA following the instructions of the cDNA synthesis with the RevertAid H Minus Reverse Transcriptase kit (Thermo Fisher Scientific). The semiquantitative PCR was performed on the Biomark HD system (Standard BioTools, San Francisco, CA, USA) using EvaGreen DNA binding Dye with BioMark 48.48 Dynamic Array Integrated Fluidic Circuits according to the manufacturer’s recommendations. All reactions were run in duplicates. Sequences of exon-spanning primers of target genes are listed below (Table 1). To quantify the relative expression, the expression of genes of interest were normalized to the mean of the reference gene Actin β (Actb). The relative change in gene expression was calculated utilizing the 2­(-Delta–Delta C­(T)) method.^33^

**Table 1:** List of Exon-Spanning Primers Used for Gene Expression Analysis in WAT

**gene**	**forward primer**	**reverse primer**
*Actb*	GACGGCCAGGTCATCACTAT	CTTCTGCATCCTGTCAGCAA
*Adipoq*	GGAACTTGTGCAGGTTGGAT	GCTTCTCCAGGCTCTCCTTT
*Cat*	CCTTCAAGTTGGTTAATGCAGA	CAAGTTTTTGATGCCCTGGT
*Cd36*	GGACATTGAGATTCTTTTCCTCTG	GCAAAGGCATTGGCTGGAAGAAC
*Fabp4*	TCACCTGGAAGACAGCTCCT	AATCCCCATTTACGCTGATG
*Fasn*	CACAGTGCTCAAAGGACATGCC	CACCAGGTGTAGTGCCTTCCTC
*Fbn1*	CAAGAGACGGAGAAGCACGA	GCAGGAGCTCTAGGATTCGG
*Furin*	TGGCATTGTGGTCTCCATCC	CCTACGCCACAGACACCATT
*Gpx8*	AAATTCCACCTTGGCTCCTT	ACATTCCCCATCTTCCACAA
*Il1b*	GTGGCAGCTACCTGTGTCTT	GGAGCCTGTAGTGCAGTTGT
*Irs1*	CAGTGTCACCCCAGATTCCC	CCTTGCCACCCATGCAGATA
*Lep*	AGAAGATCCCAGGGAGGAAA	TGAAGCCCAGGAATGAAGTC
*Lpl*	TTTGGCTCCAGAGTTTGACC	GTCTTGCTGCTGTGGTTGAA
*Pparg*	TTCAGAAGTGCCTTGCTGTG	TCCGTTGTCTTTCCTGTCAA
*Slc2a4*	GGTGTGGTCAATACGGTCTTCAC	AGCAGAGCCACGGTCATCAAGA
*Srebf1*	CGACTACATCCGCTTCTTGCAG	CCTCCATAGACACATCTGTGCC
*Tnf*	TCTTCTCATTCCTGCTTGTGG	GGTCTGGGCCATAGAACTGA

### 2.6 Statistical Analysis

All data were analyzed using GraphPad Prism 10 or R version 4.5.1 software. Normal distribution was evaluated by applying the Kolmogorov–Smirnov test for data sets with >50 samples or the Shapiro-Wilk test for data sets with ≤ 50 samples. The identification of statistical outliers was conducted via implementation of the Grubbs’ test (α = 0.05), and the resulting outliers were subsequently removed from the data set. The significance of comparisons among normally distributed data was evaluated using the one-way or two-way ANOVA test, which was followed by Šidák’s or Tukey‘s multiple comparison test for groups’ comparison (Vehicle vs BPA, Vehicle vs BP-3, Vehicle vs BPA+BP-3, BPA vs BP-3, BPA vs BPA+BP-3, and BP-3 vs BPA+BP-3). The significance of non-normally distributed data was evaluated using the Kruskal–Wallis test, which was followed by Dunn’s multiple comparison test. Significance of differences between deciles of adipocyte area was evaluated by two-way ANOVA and Tukey′s multiple comparison test. A *p*-value of less than 0.05 was deemed statistically significant. Linear regression analyses, correlation analyses, as well as correlation plots were performed with R version 4.5.1 ^34^ using the packages corrplot^35^ and plotly.^36^ The gene interaction network was created using the “STRING” database for genes analyzed by RT-PCR and used for a pairwise Spearman’s correlation matrix. STRING is designed to discover the interaction of target genes and their interactions with other genes or proteins and the basic user settings of the STRING Web site were used with a minimum required interaction score of 0.400. Graphical abstracts and schematics were created using the online tool provided by BioRender.com.

## 3 Results

### 3.1 Gestational and Lactational Exposure to BPA and BP-3 had a Sex-Specific Impact on Body Mass Development of Offspring

We first assessed the body mass of offspring after weaning until P100 and found significant differences in bw throughout adolescence (postnatal week (wk) 4 – P100) between male and female offspring, showing that males were heavier than females from wk4 onward (Supporting Information, Table S1). In males aged 8 weeks, body mass was significantly increased in BPA+BP-3 compared to BP-3-exposed males, and BPA+BP-3-exposed males also had the highest mean body mass of all groups (Figure 1A). Conversely, BPA exposure led to a significant increase of body mass in wk10 and wk12 compared to controls (vehicle) in males. BPA-exposed males were significantly heavier from wk8 to wk12 and on P100 compared to BP-3-exposed males. On P100, males exposed to BP-3 had the lowest mean body mass of all groups (Figure 1A). In summary, male offspring’s body mass was affected by gestational and lactational exposure to BPA, BP-3, and BPA+BP-3, whereas female offspring’s body mass was not significantly altered by EDC exposure throughout adolescence.

**Figure 1:**
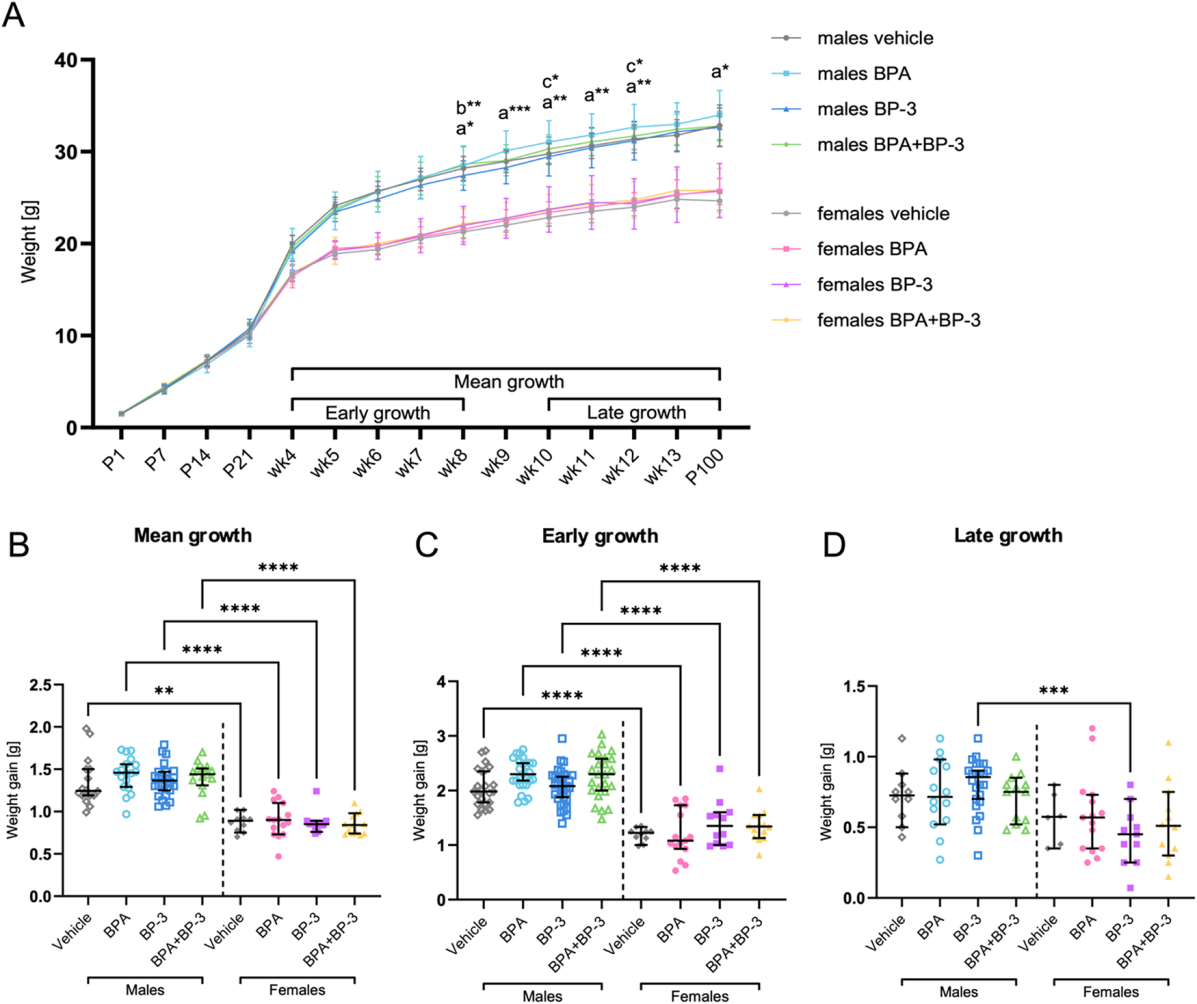
Body mass and body mass gain of male and female offspring exposed during gestation and lactation to vehicle, BPA, BP-3, and BPA+BP-3. (A) Body mass development from P1 to P100. Wk4 to 8 were categorized as early growth, wk10 to P100 as late growth, and wk4 to P100 as mean growth. A two-way ANOVA test followed by Tukey‘s multiple comparison test was used for statistical testing. The following six pairwise comparisons have been tested per sex: vehicle vs BP-3, vehicle vs BPA, vehicle vs BPA+BP-3, BPA vs BP-3, BPA vs BPA+BP-3, and BP-3 vs BPA+BP-3. Significant differences in males were a (BPA vs BP-3), b (BP-3 vs BPA+BP-3), and c (vehicle vs BPA). The number of animals (*n*) per group can be found in the Supporting Information Table S2. Details on statistical results can be found in the Supporting Information Table S3. (B) Mean growth (Kruskal–Wallis, 8 groups, statistics 80.15, *p* < 0.0001), (C) Early growth (one-way ANOVA, F (7, 147) = 33.12, *p* < 0.0001), and (D) Late growth (one-way ANOVA, F (7, 97) = 4.194, *p* = 0.0004) in male and female offspring. A one-way ANOVA followed by Šídák’s multiple comparisons test or Kruskal–Wallis test followed by Dunn‘s multiple comparison test was used for statistical testing. The following five pairwise comparisons have been tested per sex: vehicle vs BP-3, vehicle vs BPA, vehicle vs BPA+BP-3, BPA vs BPA+BP-3, and BP-3 vs BPA+BP-3. In addition, four pairwise comparisons have been tested for male vs female: vehicle vs vehicle, BPA vs BPA, BP-3 vs BP-3, and BPA+BP-3 vs BPA+BP-3. * *p* < 0.05, ** *p* < 0.01, *** *p* < 0.001, **** *p* < 0.0001. BP-3, benzophenone-3; BPA, bisphenol A; P, *p*ostnatal day; wk, postnatal week.

Next, we were interested in average body mass gain during adolescence, from prepubescent age (wk4) until sexual maturity (wk8), and average body mass gain after reaching sexual maturity (wk10) until P100, representing early adulthood.^37^ Thus, we defined early growth as average body mass gain in grams per week from wk4 to wk8, late growth as average body mass gain in grams per week from wk10 to P100, and mean growth as average body mass gain in grams per week from wk4 to P100 (Figure 1B–D). We found that males of all exposure groups had a significantly higher early and mean growth compared to females (Figure 1B,C). Moreover, males exposed during gestation and lactation to BP-3 had a significantly higher late growth compared with females exposed to BP-3. In fact, the BP-3-exposed males had the highest average late growth, whereas BP-3-exposed females showed the lowest late growth (Figure 1D). Conversely, males and females exposed during gestation and lactation to BPA had the highest average mean growth from week 4 to P100 (Figure 1B). These data suggest a significant impact of gestational and lactational exposure to BPA during late adolescence and early adulthood and to BP-3 during early adulthood in male body mass, as well as sex-specific differences in body mass and late growth following gestational and lactational EDC exposure.

### 3.2 Gestational and Lactational Exposure to BPA and BP-3 Resulted in Sex-Specific Morphological Changes in WAT of the Offspring

Morphological changes of WAT were assessed by measuring alterations of adipocyte area by histological means. When comparing the mean adipocyte area, the most pronounced effect was observed in BPA-exposed males. Adipocytes in this group were approximately 22% smaller than those in the control group, with a significant decrease of their mean area from 1396 μm^2^ in controls to 1092 μm^2^ in BPA-exposed males (Figure 2A). Equally, a significant reduction of cell size by almost 20% was detected in males exposed to a BPA+BP-3 mixture in comparison to controls. No differences in adipocyte area were observed in males exposed exclusively to BP-3 compared to controls. Subsequently, a significant decrease of adipocyte area was found when comparing BP-3-exposed to BPA+BP-3-exposed males (Figure 2A). For females, no significant differences in adipocyte area were observed across all three exposure groups (Figure 2B). However, adipocyte area in the vehicle, BP-3- and BPA+BP-3-exposed females, was significantly smaller than in males of the respective exposure group (Figure 2C). Thus, adipocytes of BPA-exposed males and females were similar in size. These results indicate that gestational and lactational exposure to BPA and BPA+BP-3 significantly affect male WAT morphology, leading to smaller adipocytes, and BPA exposure in males may obliterate sex-specific morphological differences in adipocyte area.

**Figure 2:**
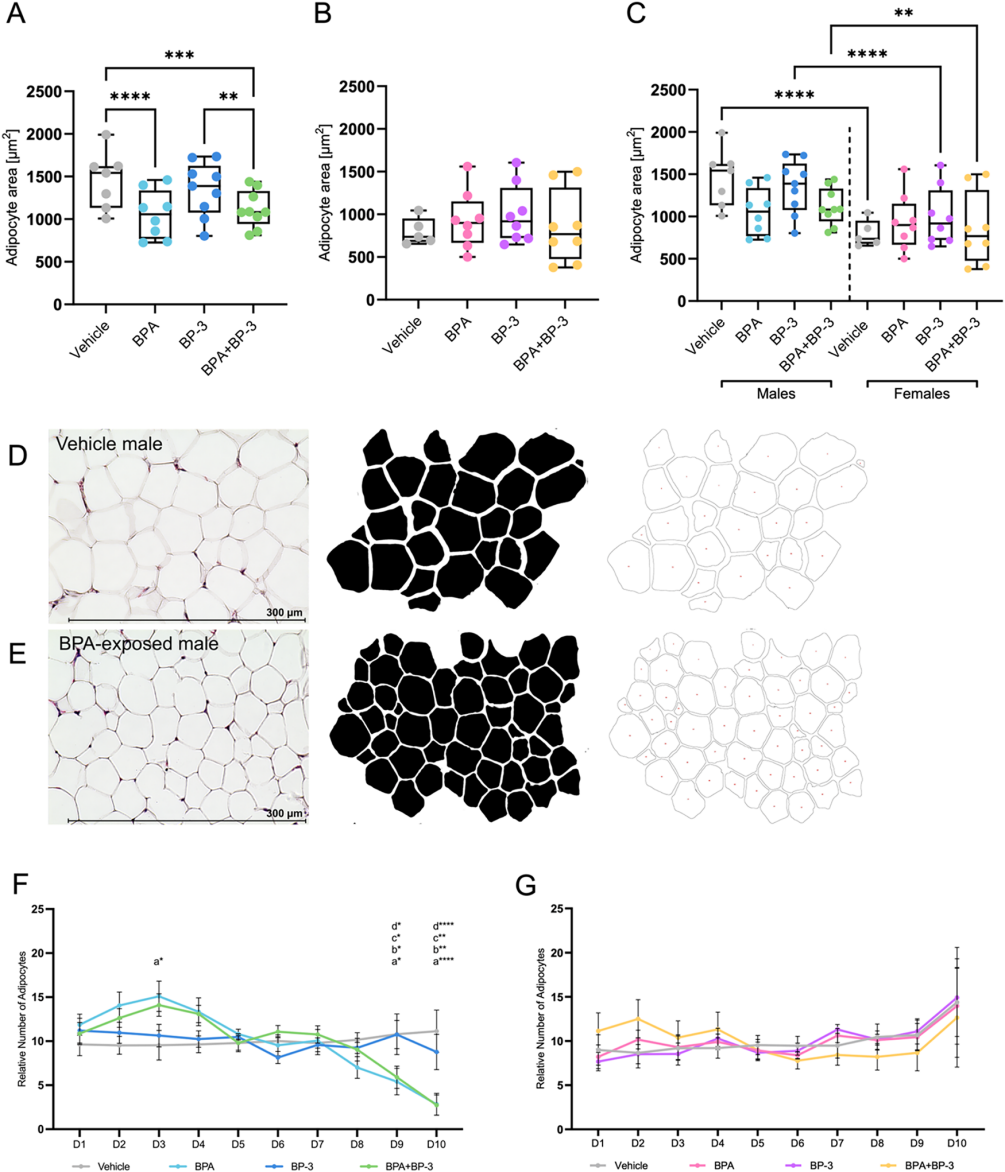
Morphological changes in WAT as a result of gestational and lactational exposure to BPA, BP-3, and BPA+BP-3. Mean adipocyte area of WAT comparing male (A) vehicle (*n* = 7), BPA (*n* = 8), BP-3 (*n* = 9), and BPA+BP-3 (*n* = 9) tested by two-way ANOVA (F (3, 157) = 12.52, *p* < 0.0001) and female (B) vehicle (*n* = 5), BPA (*n* = 8), BP-3 (*n* = 8), and BPA+BP-3 (*n* = 8) offspring tested by two-way ANOVA (F (3, 135) = 1.601, *p* = 0.1921) as well as both sexes (C) tested by two-way ANOVA (F (7, 299) = 15.6, *p* < 0.0001). A two-way ANOVA test followed by Šídák’s multiple comparisons test was used for statistical testing. The following five pairwise comparisons have been tested: vehicle vs BP-3, vehicle vs BPA, vehicle vs BPA+BP-3, BPA vs BPA+BP-3, and BP-3 vs BPA+BP-3. Representative images of H/E stained WAT sections (40× magnification, scale bar = 300 μm) of (D) male vehicle and (E) male BPA-exposed group and the extracted area of adipocytes using ImageJ. The relative number of adipocytes per decile, which represent 10% of all adipocytes in the vehicle group (gray line) for male (F) vehicle (*n* = 7), BPA (*n* = 8), BP-3 (*n* = 9), and BPA+BP-3 (*n* = 9) and female (G) vehicle (*n* = 6), BPA (*n* = 8), BP-3 (*n* = 8) and BPA+BP-3 (*n* = 8). A two-way ANOVA test followed by Tukey‘s multiple comparison test was used for statistical testing. The following six pairwise comparisons have been tested per sex: vehicle vs BP-3, vehicle vs BPA, vehicle vs BPA+BP-3, BPA vs BP-3, BPA vs BPA+BP-3, and BP-3 vs BPA+BP-3. Significant differences were a (vehicle vs BPA), b (BPA vs BP-3), c (BP-3 vs BPA+BP-3), and d (vehicle vs BPA+BP-3). * *p* < 0.05, ** *p* < 0.01, *** *p* < 0.001, **** *p* < 0.0001. More Details on statistical results for Figure 2F,G can be found in the Supporting Information Table S5.

### 3.3 Gestational and Lactational BPA and BP-3 Exposure Induced a Shift in Adipocyte Area Distribution, Favoring Small to Medium-Sized Cells in Males

To more accurately assess the phenotypic shift of the observed changes in adipocyte area in males, the entirety of adipocytes from control animals were sorted by area and divided into deciles, each representing 10% of the total adipocyte population (Supporting Information, Table S4). The margins of each decile, determined from the control group, were then applied to individual exposure groups. This approach enabled the creation of a figure illustrating the distribution of adipocyte areas across the four groups (Figure 2F,g).

Differences in adipocyte area distribution were observed in male offspring following gestational and lactational BPA and BP-3 exposure (Figure 2F). A significant increase in adipocyte number was observed in decile 3 (D3), ranging in area from 491 to 758 μm^2^ per adipocyte, in BPA-exposed males. In this decile, 15.1% of adipocytes were detected instead of the expected 10% found in D3 of the control group. A similar pattern was observed in animals exposed to a BPA+BP-3 compared to controls (not significant). In decile D10, representing adipocyte area of 2660 μm^2^ and larger, the relative number of adipocytes per decile in BPA- and BPA+BP-3-exposed males decreased significantly by >70% compared to controls and >50% compared to BP-3-exposed males. Decile 9 showed significant results of the same pattern, but not as pronounced. Among males, adipocyte distribution across deciles did not significantly differ from that of the control group only for the BP-3-exposed group. In contrast, for females, no significant differences in the distribution of adipocytes were detected among EDC-exposed and control groups. Therefore, our results suggest that gestational and lactational BPA and BPA+BP-3 exposure in males, but not in females, may induce a shift toward adipocyte hyperplasia rather than hypertrophy.

### 3.4 BPA and BP-3 Exposure Influenced the Expression of Genes Regulating Adipogenesis and Adipokine Signaling in WAT in a Sex-Specific Manner

Given that previous investigations showed changes in animal body mass and WAT morphology, we aimed to characterize the functional changes and underlying mechanisms in adipocytes following gestational and lactational exposure to BPA and BP-3. Therefore, the expression of genes involved in adipogenesis (Pparg, Fabp4, Srebf1), lipid turnover (Lpl, Fasn, Cd36), and glucose metabolism (Irs1, Slc2a4) was quantified by RT-PCR in WAT. Moreover, genes of adipokine signaling involved in endocrine regulation of WAT (Lep, Adipoq, Asprosin (Fbn1)) were measured as well as genes of antioxidant enzymes (Cat, Gpx8) and markers for tissue inflammation (Tnf, Il1b, Il6).

The results showed a significant upregulation of Pparg in the WAT of male offspring exposed to the BPA+BP-3 mixture (Figure 3A) and downregulation (not significant) of Pparg in female offspring exposed to BP-3 compared to controls (Figure 3B). Increases in Fabp4, Cd36, Irs1 (Figure 3E–P), Fasn, and Lpl (Figure 4A,C) were also observed in both the BPA+BP-3- and BPA-exposed males, along with elevated Lep (Figure 3I) and Slc2a4 levels (Figure 3M) in the BPA-exposed males compared to controls, and these increases were not statistically significant. In females, exposure to BP-3 caused a significant downregulation of Fabp4 and Adipoq gene expression compared to controls (Figure 3D,H), and Lep gene expression was lower in BP-3-exposed females compared to controls without reaching statistical significance (Figure 3J). Moreover, no significant differences were observed in the expression of antioxidant enzyme or inflammation marker genes in males or females, but the same sex-dependent trends could be observed for Cat (Figure 4N). These results suggest that there are sex-specific differences in the regulation of adipogenesis and adipokine gene expression following gestational and lactational BPA and BP-3 exposures.

**Figure 3:**
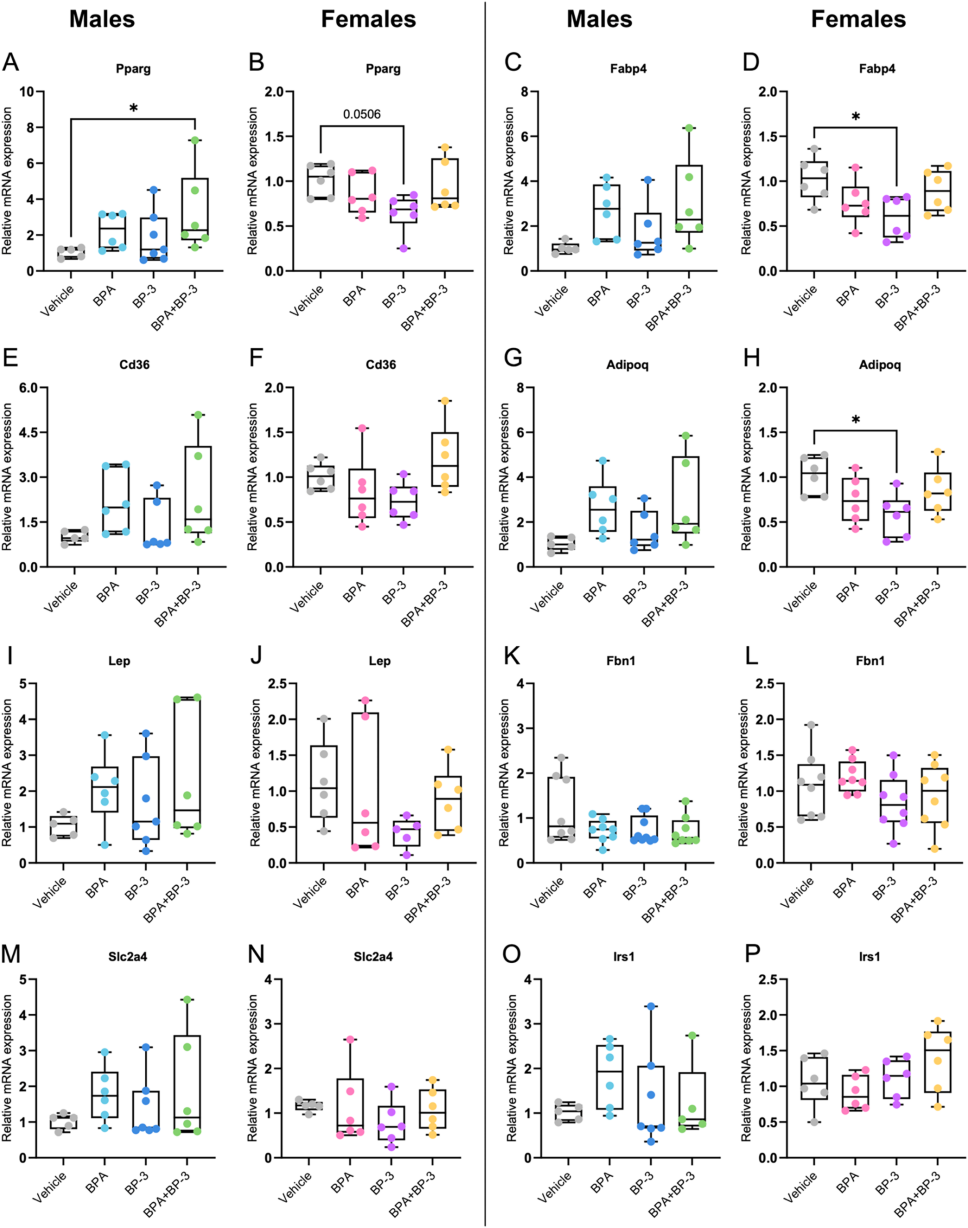
Gene expression of adipogenesis, adipokine, and glucose metabolism markers in male and female offspring exposed to BPA and BP-3 during gestation and lactation. Relative mRNA expression of Pparg in males (Kruskal–Wallis, 4 groups, statistics 8.158, *p* = 0.0429) (A) and females (B), Fabp4 in males (C) and females (one-way ANOVA (F (3, 20) = 3.598, *p* = 0.0316)) (D), Cd36 in males (E) and females (F), Adipoq in males (G) and females (one-way ANOVA (F (3, 20) = 3.497, *p* = 0.0346)) (H), Lep in males (I) and females (J), Fbn1 (Asprosin) in males (K) and females (L), Slc2a4 in males (M) and females (N), and Irs1 in males (O) and females (P). Each dot per box plot represents one animal after removal of statistical outliers. A one-way ANOVA followed by Šídák’s multiple comparisons test or Kruskal–Wallis test followed by Dunn‘s multiple comparison test was used for statistical testing. The following five pairwise comparisons have been tested per sex: vehicle vs BP-3, vehicle vs BPA, vehicle vs BPA+BP-3, BPA vs BPA+BP-3, and BP-3 vs BPA+BP-3. * *p* < 0.05.

**Figure 4:**
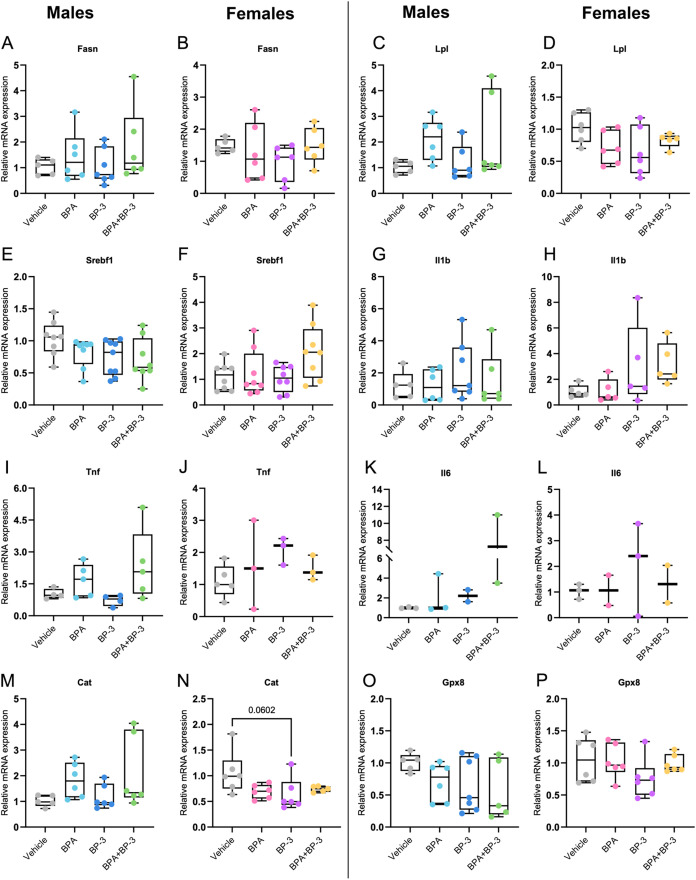
Gene expression of lipid metabolism, inflammation, and oxidative stress markers in male and female offspring exposed to BPA and BP-3 during gestation and lactation. Relative mRNA expression of Fasn in males (A) and females (B), Lpl in males (C) and females (D), Srebf1 in males (E) and females (F), Il1b in males (G) and females (H), Tnf in males (I) and females (J), Il6 in males (K) and females (L), Cat in males (M) and females (N), and Gpx8 in males (O) and females (P). Each dot per box plot represents one animal after removal of statistical outliers. A one-way ANOVA followed by Šídák’s multiple comparisons test or Kruskal–Wallis test followed by Dunn‘s multiple comparison test was used for statistical testing. The following five pairwise comparisons have been tested per sex: vehicle vs BP-3, vehicle vs BPA, vehicle vs BPA+BP-3, BPA vs BPA+BP-3, and BP-3 vs BPA+BP-3.

### 3.5 Associations between Gene Expression, Adipocyte Area, and Growth Differed Depending on Offspring Sex and EDC Exposure, and the STRING Network Revealed Target Gene Interaction

Since we found sex-specific differences in EDC-exposed offspring in body mass, growth, adipocyte area, and gene expression of specific marker genes in WAT, we performed correlation analysis to understand the relationship between these variables in a sex-dependent manner, taking gestational and lactational EDC exposure into account. We found that adipocyte area was positively associated with early growth, mean growth, and body mass on P56 (wk8) in BP-3-exposed male offspring; however, in BPA+BP-3-exposed males, early and mean growth were negatively associated with adipocyte area (Figure 5). In BPA-exposed males, we observed a significant negative association between adipocyte area and early, mean, and late growth and body mass on P100 and these associations were almost absent in the vehicle group. In female offspring, vehicle, BP-3, and BPA+BP-3 exposure showed a positive association between adipocyte area, late and mean growth, as well as body mass on P100 (Figure 6). Because of a high number of missing values in vehicle- and BPA-exposed females for certain genes, these matrices are missing. In males exposed to BPA+BP-3, expression of most genes was positively associated with adipocyte area, except for furin and Srebf1, whereas in BP-3-, BPA-, and vehicle-exposed males, these associations were mostly negative or not significant (Figure 5). Early growth showed negative associations to gene expression of most analyzed marker genes in vehicle- and BPA+BP-3-exposed males, and these associations were not observed in BPA- and BP-3-exposed males. Gene expression of Lep and Pparg was strongly positively associated with adipocyte area and negatively associated with early and mean growth in males exposed to BPA+BP-3. These positive associations of Lep and Pparg gene expression with adipocyte area were contrary or not significant in males exposed to BPA, BP-3, or vehicle. However, several genes were significantly negatively associated with early and mean growth in BPA+BP-3- and vehicle-exposed males, except for Gpx8, Il1b, and Tnf. Moreover, these genes showed different association patterns in BPA, BP-3, and BPA+BP-3 exposure in both sexes. In females exposed to vehicle, BP-3, and BPA+BP-3, adipocyte area was positively associated with late and mean growth and body mass on P100; however, the positive association in BP-3-exposed females between adipocyte area and gene expression of Lep, Lpl, and Tnf was not significant or absent in BPA+BP-3- and vehicle-exposed females (Figure 6). Furthermore, in BP-3-exposed females, early growth was significantly positively associated with Fasn, Irs1, Pparg, and Slc2a4 gene expression, but these associations were negative or not significant in vehicle- and BPA+BP-3-exposed females. In summary, these results indicate that there are specific associations of gene expression in WAT, adipocyte area, body mass, and growth in males and females, and these sex-specific associations are altered following gestational and lactational EDC exposure.

**Figure 5:**
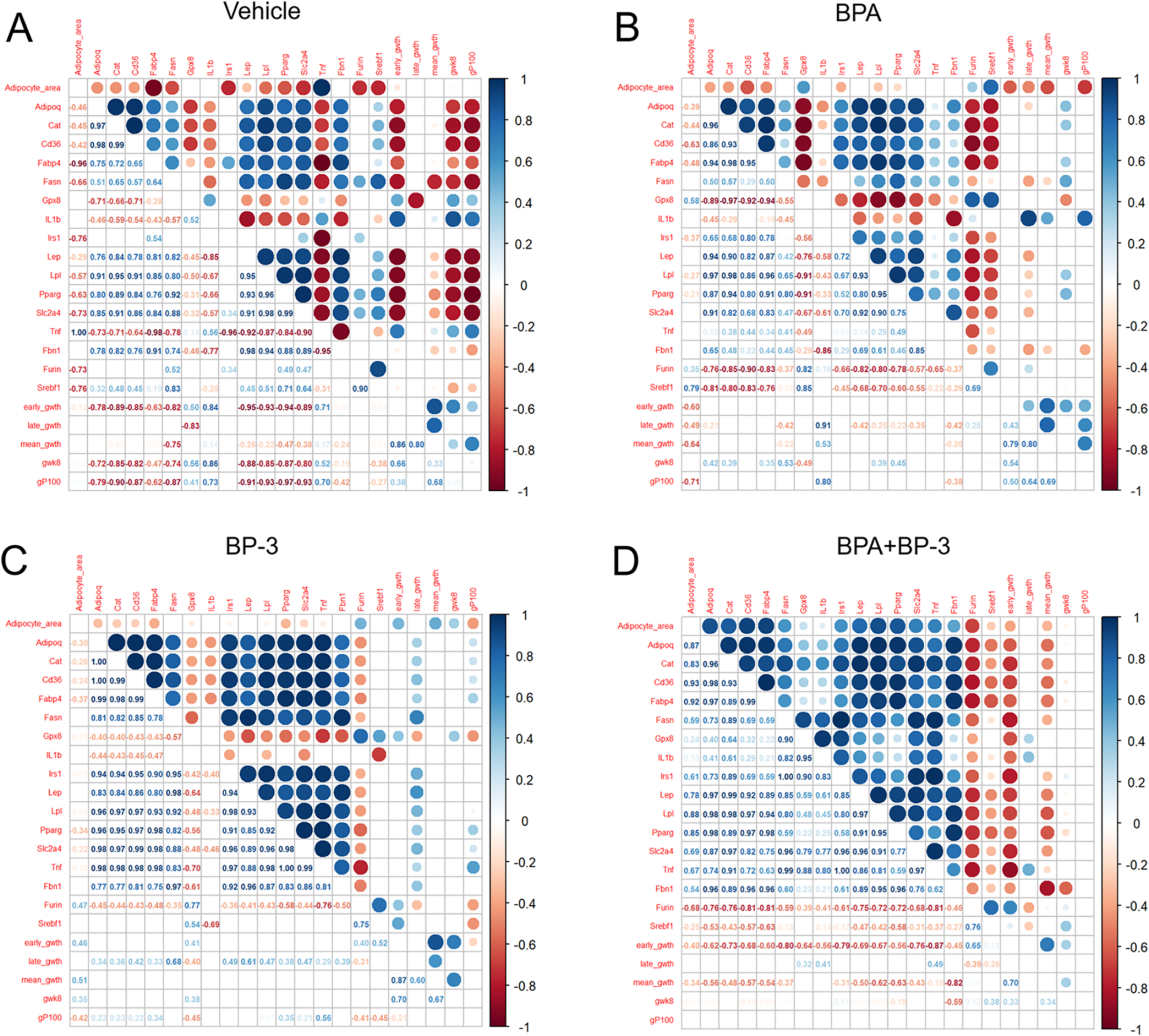
Pairwise Spearman’s correlation matrix of adipocyte area, gene expression, and growth in male offspring exposed to BPA and BP-3 during gestation and lactation. The plots show data from male offspring exposed to (A) vehicle, (B) BPA, (C) BP-3, and (D) BPA+BP-3. The color spectrum indicates positive correlations in blue and negative correlations in red between the parameters. The color intensity displays the correlation coefficient magnitude. Empty fields represent nonsignificant correlations. early_gwth = early growth, late_gwth = late growth; mean_gwth = mean growth; gwk8 = body mass at postanal week 8; gP100 = body mass on postnatal day 100.

**Figure 6:**
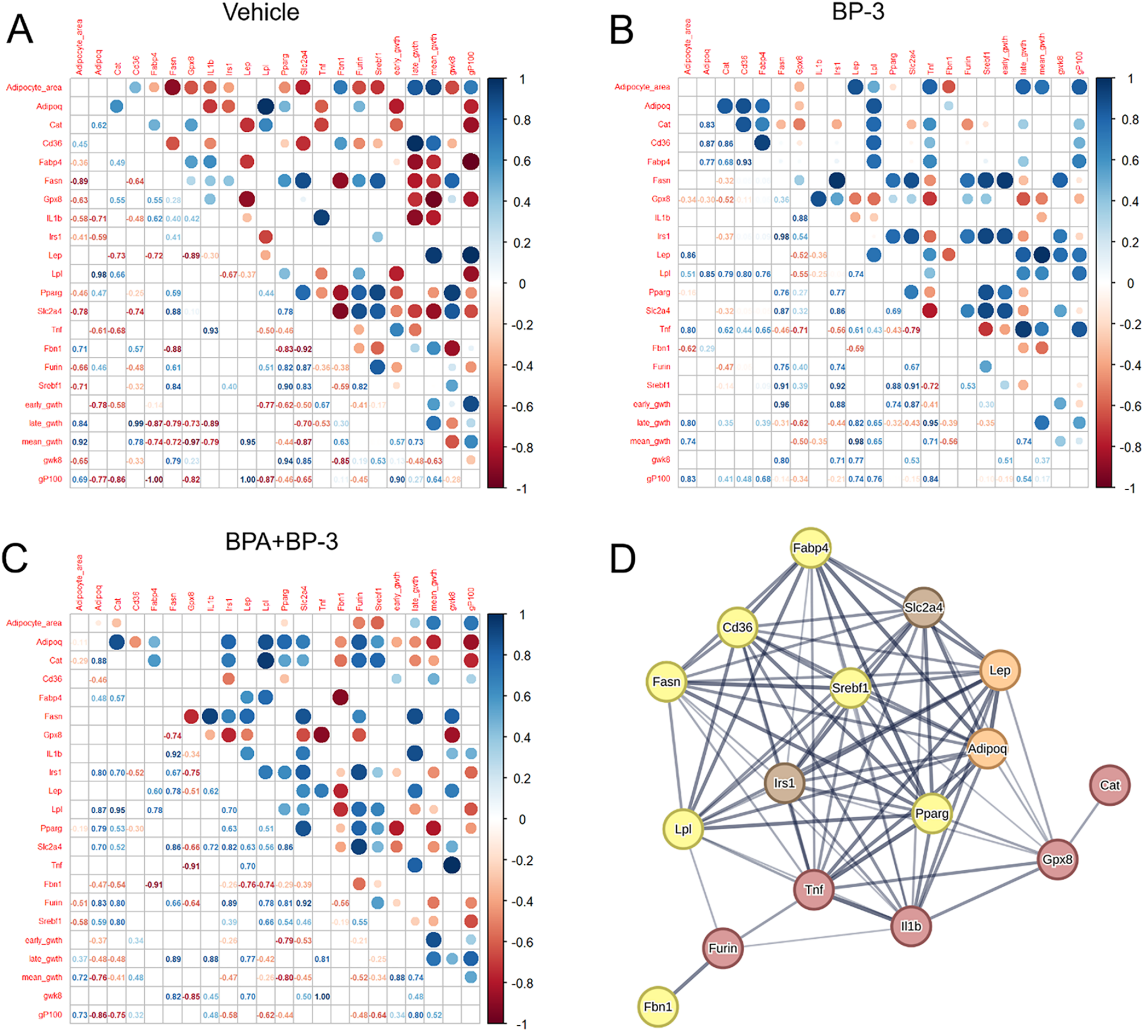
Pairwise Spearman’s correlation matrix of adipocyte area, gene expression, and growth in female offspring exposed to BPA and BP-3 during gestation and lactation. The plots show data from female offspring exposed to (A) vehicle, (B) BP-3, and (C) BPA+BP-3. The color spectrum indicates positive correlations in blue and negative correlations in red between the parameters. The color intensity displays the correlation coefficient magnitude. Empty fields represent nonsignificant correlations. (D) STRING interaction networks show the genes analyzed by RT-PCR in male and female WAT and used for a pairwise Spearman’s correlation matrix. Yellow nodes show genes of adipogenesis and lipid turnover, red nodes show genes of tissue inflammation markers and antioxidant enzymes, orange nodes show genes of endocrine regulation (adipokines), and brown nodes show genes of glucose metabolism. STRING is designed to discover the interaction of target genes and their interactions with other genes or proteins and the basic user settings of the STRING Web site were used with a minimum required interaction score of 0.400 (thin lines), 0.700 (medium lines), and 0.900 (thick lines). early_gwth = early growth, late_gwth = late growth; mean_gwth = mean growth; gwk8 = body mass at postanal week 8; gP100 = body mass on postnatal day 100.

Besides the association between gene expression, adipocyte area, and growth, we were interested in the associations of gene expression of marker genes with each other. To analyze the interactions of our gene set comprising 16 genes related to adipogenesis (Pparg, Fabp4, Srebf1), lipid turnover (Lpl, Fasn, Cd36), glucose metabolism (Irs1, Slc2a4), endocrine regulation (Lep, Adipoq, asprosin (Fbn1)), antioxidant enzymes (Cat, Gpx8), and markers for tissue inflammation (Tnf, Il1b), these genes were used to create a STRING interaction network for both sexes (Figure 6D). Functional enrichment analysis with STRING showed that selected genes were involved in the regulation of hormone secretion, response to fatty acid, fatty acid binding, as well as AMPK (AMP-activated protein kinase) and adipocytokine signaling pathways. As anticipated based on scientific literature, STRING revealed interactions between Cat and Gpx8, Fbn1 and Furin, as well as Pparg and Fabp4, Srebf1, Fasn, and Irs1 (Figure 6). However, a positive association between Cat and Gpx8 gene expression in WAT was only found in BPA+BP-3-exposed males, whereas Fbn1 and Furin gene expression showed a negative association with each other in EDC-exposed but not in vehicle-exposed males (Figure 5). Pparg gene expression was positively associated with Fabp4, Srebf1, and Fasn in EDC- and vehicle-exposed males, contrary to Irs1 gene expression, which was positively associated with Pparg gene expression in EDC-exposed, but not in vehicle-exposed males. Moreover, Irs1 gene expression was positively associated with Cd36 and Slc2a4 and negatively associated with Srebf1 gene expression in BPA+BP-3- and BPA-exposed males, which was not observed in BP-3- and vehicle-exposed males. In females exposed to BPA+BP-3, BP-3, and vehicle, associations of Irs1 gene expression with Cd36 and with Srebf1 gene expression were contrary to these same associations in males (Figure 6). Interestingly, in BPA+BP-3-exposed males, gene expression of Adipoq, Lep, Il1b, Tnf, Cat, and Gpx8 showed positive associations with each other, whereas the association of Adipoq gene expression with Gpx8 and Il1b was negative in BPA-, BP-3-, and vehicle-exposed males. Thus, associations of gene expression of known interacting genes in adipogenesis, lipid turnover, glucose metabolism, oxidative stress, and inflammation are different in EDC-exposed offspring of both sexes and for different chemicals.

## 4 Discussion

The current study was performed by using a mouse model of gestational and lactational EDC exposure with environmentally relevant concentrations. This was achieved by applying BP-3 to mice at a similar dose as applied by humans using sunscreen products^32^ and BPA within the TDI range as set by the EFSA in 2015. In 2023, during the conduct of this study, the EFSA announced that the TDI of BPA should be reduced to 0.2 ng/kg/d, i.e., by a factor of 20,000 compared to the TDI of 2015.^22^ However, a decrease in BPA exposure in accordance with the new TDI is rather unlikely to occur as a consequence of the ubiquitous presence of BPA in the environment and the ongoing use of BPA and its structural analogues in consumer products from outside the EU.^38^ In addition to studying the effects of applying BPA and BP-3 as a single exposure, the combined effects of both of these chemicals as an EDC mixed exposure were investigated. Single EDC substances have been studied multiple times for their endocrine effects in vivo and in vitro; however, there is still a significant shortage of in vivo studies investigating mixture effects. It is worth mentioning that our mouse model of gestational and lactational exposure to EDCs comprised offspring of a mixed background strain, leading to heterozygous BALB/c-C57BL/6 offspring. This mixed background has the advantage of being more comparable to a heterozygous individual, thereby ameliorating mouse strain and gene-related bias for certain metabolic or immune phenotypes.^39^

We previously reported that prenatal BP-3 exposure affected intrauterine development of male and female offspring leading to reduced body mass and growth restricted fetuses compared to vehicle exposure.^40^ Postnatally, female offspring exposed to BPA+BP-3 were significantly more often large-for-gestational-age on P1, had the highest mean body mass on P1 to P21, and were significantly heavier than vehicle controls on P7.^31^ We also found that male offspring exposed to BPA+BP-3 were more frequently large-for-gestational-age pups on P1 and were significantly heavier than controls on P7 and P21 (unpublished data). In the current study, the goal was to investigate the body mass and growth of offspring exposed to EDCs during gestation and lactation after weaning until adulthood. We found that gestational and lactational exposure to BPA, BP-3, or BPA+BP-3 affected body mass gain and body mass at different points in time during pubertal and postpubertal adolescence in males. However, adipocytes of BPA- and BPA+BP-3-exposed males were significantly smaller compared to vehicle- and BP-3-exposed males. Thus, the mechanism for increased body mass in both BPA- and BPA+BP-3-exposed males is not related to adipocyte hypertrophy and may be related to adipocyte hyperplasia, which is supported by a higher count of small adipocytes and increased Pparg gene expression compared to vehicle- and BP-3-exposed males. Alternative mechanisms that could explain increased body mass, such as increased fat-free mass, body length, or hyperphagia, were not evaluated in this study. However, male offspring exposed to BP-3 had the highest growth or body mass gain per week after week 10 to P100, and they had similar sized adipocytes as the vehicle group, which may suggest a general increase in body mass rather than adipocyte hypertrophy. Based on our previous findings of an intrauterine growth restriction phenotype in males exposed to BP-3,^40^ we speculate that there might be a predisposition or perinatal programming, possibly caused by epigenetic mechanisms, for an increased growth to “catch-up” for an intrauterine developmental delay following in utero BP-3 exposure. There is evidence in the literature that perinatal exposure to EDCs like BP-3 can reduce fetal growth and body mass,^41–43^ and it is also known that these physiological changes can have lifelong effects for the metabolism and the risk to develop metabolic diseases.^44–46^ It also stands to reason that gestational and lactational exposure to EDCs has a more pronounced impact on the adipose tissue of male rather than female offspring. Since gene expression of Pparg, Fabp4, and Adipoq was downregulated in WAT of BP-3-exposed females compared to controls, these animals may counteract the predisposition to a “catch-up” growth, making the EDC-induced catch-up growth sex-specific to males. Thus, the observed physiological and gene expression changes in male offspring WAT following EDC exposure may be a concern for long-term health, and future studies should investigate how different forms of lifestyle or diet can further exaggerate these physiological changes toward metabolic diseases in both sexes.

Transgenerational effects of BPA exposure during early life have been described by several studies. Perinatal exposure in particular can leave an impact on gene imprinting across several generations.^47^ The adipose tissue of rodents exposed to BPA during gestation and lactation and dissected on day 21 was found to be heavier with hypertrophic adipocytes and hyperlipidemia, hypercholesterolemia, as well as increased expression for lipogenic genes.^48,49^ In rats, postnatal (lactational) exposure to high doses of BPA (5 mg/kg/d) led to a significant increase in female, but significant decrease in male visceral fat mass on P15 compared to controls, highlighting sex-specific effects of BPA albeit at significantly higher dosing.^50^ Moreover, in vitro studies have confirmed BPA’s ability to trigger WAT expansion via activation of PPARg, a master regulator of adipogenesis.^51^ Concerning human cohort research, there have been differing results, with some studies documenting a positive correlation between childhood obesity and early life exposure to BPA.^52^ However, none of the mentioned studies monitored patients’ follow-up body mass data up to adulthood. A particular study focusing on BP-3′s impact on murine mammary glands revealed significantly hypertrophic adipocytes in adulthood after perinatal exposure.^53^ Importantly, BP-3 and its metabolites have presented disrupted neuronal PPARg signaling^54^ as well as estrogenic activity in vitro, suggesting a possible interference in endocrine regulatory pathways.^55^ Additionally, human data support this hypothesis by correlating BP-3 exposure to menstrual cycle hormonal abnormalities and an increased risk of uterine pathologies.^56^

Previously, C57BL/6J mice have been shown to develop hypertrophic adipocytes within just 3 days of switching to a high-fat diet.^57^ Moreover, adipose tissue expansion was followed by the onset of insulin resistance in the same mouse strain.^58^ Therefore, we assume that exposure to environmental pollutants might result in adipose tissue hyperplasia and subsequently hypertrophy when paired with a common Western-style diet rich in saturated fats, hence multiplying the risk of obesity-associated complications. The fact that we did not observe such significant differences in adipocyte size, body mass, and growth in female offspring exposed during gestation and lactation to EDCs may be explained by a different regulation of growth and adipocyte development, possibly under the influence of adipogenesis genes, like Pparg, Fabp4, and others, and hormones. Changes in hormone receptor gene expression in the placenta of females, such as progesterone and estrogen receptors, were not observed in males^40^ and may support different hormonal regulation in both sexes mediated by the placenta before birth. Also, expression of genes related to adipogenesis, adipokines, and metabolism, such as Pparg, Fabp4, Adipoq, and Lpl, was downregulated in WAT of females exposed to BPA and BP-3. These results suggest that endocrine differences in females may attenuate EDC-mediated effects on adipose tissue hyperplasia following gestational and lactational EDC exposure. However, it is worthwhile to further investigate potential metabolic health risks in EDC-exposed females in combination with different lifestyles and diets.

WAT is an important endocrine organ that regulates lipid metabolism and storage of lipids and can also contribute to inflammation.^59,60^ We analyzed the gene expression of genes coding for antioxidant enzymes and markers of tissue inflammation. Interestingly, we did not find significant upregulation of markers of inflammation or antioxidant enzymes, suggesting that gestational and lactational exposure to EDCs did not cause WAT inflammation or enhanced oxidative stress. In fact, several animals may have had very low mRNA levels of Tnf, Il1b, and Il6, resulting in nonquantifiable RT-PCR products after preamplification with different primers. However, our study has the limitation of high variability in gene expression between animals, which may be related to the genetic background and heterozygosity, and missing data points in certain analyses that also limit the conclusions that can be drawn from the results. Knowing about the interindividual variation of this model, increasing the number of samples could yield higher statistical power.

Since complex molecular pathways determine physiological functions, and changes in the expression of one gene may trigger additional changes in other genes of the same and other pathways, it is worthwhile to investigate the relationship or interaction of multiple genes and pathways.^61^ Using the STRING database, we could show that the 16 genes chosen for analysis indeed have various interactions with each other and form a complex network. With Pparg as one of the central regulators of adipogenesis,^62^ it becomes evident that an upregulation in gene expression of Pparg, as observed in BPA+BP-3-exposed males, may trigger changes in gene expression of related genes in certain pathways, leading to adipocyte hyperplasia. Similarly, downregulation of Fabp4 and its possible interaction partners Pparg and Adipoq in females exposed to EDCs like BP-3 during gestation and lactation may explain the absence of hyperplasia and limited adipogenesis.^63,64^ In males exposed to BPA+BP-3, adipocyte area was significantly positively correlated with gene expression of 14 out of 16 chosen genes, involved in the regulation of hormone secretion, response to fatty acid, fatty acid binding, as well as AMPK and adipocytokine signaling pathways, according to STRING network analysis. These correlations were not observed in other exposure groups, vehicle controls, or female offspring, suggesting specific changes to adipocyte physiology following a combined BAP+BP-3 exposure in males. One limitation of the correlation analyses that needs to be addressed is the number of missing values, especially in female offspring data sets. These are the results of a small sample size of female offspring, which makes the correlation analysis an explorative approach rather than hypothesis testing, and consequently, the results should be interpreted with modesty.

## 5 Conclusion

Taken together, gestational and lactational exposure to BPA, BP-3, and a BPA+BP-3 mixture leads to sex-specific effects in postnatal body mass, growth, and WAT morphology, causing adipocyte hyperplasia after BPA and BPA+BP-3 exposure and a catch-up growth after BP-3 exposure only in male offspring. Expression of genes involved in adipogenesis, adipokines, and metabolism suggests Pparg as a possible mediator for WAT hyperplasia in BPA- and BPA+BP-3-exposed males and Fabp4, Adipoq, and Lpl as possible mediators to counteract BP-3-induced dysregulation of body mass during adulthood in females. These sex-specific differences should be considered when addressing preventive and therapeutic strategies for metabolic diseases in the future, in addition to studying the metabolic effects of pre- and postnatal exposure to EDC mixtures.

## Supplementary Material



## Notes

Summary data of body mass of male and female offspring exposed to BPA and BP-3 during gestation and lactation; number of male and female offspring per exposure group; statistical results of Two-way ANOVA for body mass analysis of EDC-exposed offspring from P1 to P100; number of adipocytes included in each decile and their respective minimal and maximal area; statistical results of Two-way ANOVA for adipocyte area distribution across deciles (PDF)
