# Supplementary material for: Combined Exposure to Endocrine Disruptors, BPA and BP-3, during Pregnancy and Lactation Alters Postnatal Body Mass, Growth, White Adipose Tissue Morphology, and Adipogenic Gene Expression in a Sex-Specific Manner
Source: Environ Health (Wash). 2026 Apr 20;4(7):1373–87. doi: 10.1021/envhealth.5c00679 (PMC13386372; doi:10.1021/envhealth.5c00679)
Supplement: Supplementary file 1 [file eh5c00679_si_001.pdf]

Supporting Information

Combined exposure to endocrine disruptors BPA and BP-3 during pregnancy and lactation alters postnatal body mass, growth, white adipose tissue morphology and adipogenic gene expression in a sex-specific manner

*Kristína Raticová[1,2], Julia Howanski[1,2], Beate Fink[1], Stefan Röder[1], Mario Bauer[1], Anne Schumacher[1,2,3], Ana C. Zenclussen[1,2,3,4,5], Tobias Kretschmer[1,2,3,5]**

[1] Department of Environmental Immunology, Helmholtz Centre for Environmental Research - UFZ, Leipzig, Germany.

[2] Perinatal Immunology, Saxonian Incubator for Clinical Translation (SIKT), Medical Faculty, Leipzig University, Leipzig, Germany.

[3] Leipzig Reproductive Health Research Center (LE-REP), Leipzig University, Leipzig, Germany.

[4] German Center for Child and Adolescent Health (DZKJ), Partner Site Leipzig/Dresden, Germany.

[5] LeiCeM - Leipzig Center of Metabolism, Leipzig University, Leipzig, Germany.

* Corresponding author: Dr. Tobias Kretschmer, electronic address: tobias.kretschmer@ufz.de

Table S1. Summary data of body mass of male and female offspring exposed to BPA and BP-3 during gestation and lactation. The table shows minimum and maximum body mass (Min / Max), median body mass with interquartile range (Med [IQR]), mean body mass with standard deviation (Mean (SD)) and the total number of animals per sex (*n*). wk = postnatal week. P100 = Postnatal day 100.

Label	Variable	Sex		Test
		f	m	
4wk	Min / Max	14.0 / 20.4	10.9 / 23.9	*p* value: <0.0001
	Med [IQR]	16.5 [15.9;17.2]	19.4 [18.7;20.1]	(Wilcoxon rank sum test)
	Mean (SD)	16.6 (1.2)	19.5 (1.6)	
	n	51 (0)	104 (0)	
5wk	Min / Max	16.7 / 22.8	15.5 / 27.2	*p* value: <0.0001
	Med [IQR]	19.1 [18.8;19.6]	23.8 [23.1;24.3]	(Wilcoxon rank sum test)
	Mean (SD)	19.2 (1.1)	23.7 (1.4)	
	n	51 (0)	104 (0)	
6wk	Min / Max	17.9 / 24.2	17.9 / 30.7	*p* value: <0.0001
	Med [IQR]	19.5 [19.1;20.0]	25.4 [24.5;26.3]	(Wilcoxon rank sum test)
	Mean (SD)	19.7 (1.1)	25.4 (1.7)	
	n	51 (0)	104 (0)	
7wk	Min / Max	18.7 / 26.4	20.0 / 32.3	*p* value: <0.0001
	Med [IQR]	20.5 [20.1;21.2]	26.9 [25.8;27.8]	(Wilcoxon rank sum test)
	Mean (SD)	20.8 (1.4)	26.9 (1.8)	
	n	51 (0)	104 (0)	
8wk	Min / Max	19.1 / 27.7	21.3 / 32.7	*p* value: <0.0001
	Med [IQR]	21.6 [20.7;22.4]	28.1 [27.0;29.1]	(Wilcoxon rank sum test)
	Mean (SD)	21.8 (1.6)	28.1 (1.9)	
	n	51 (0)	104 (0)	
9wk	Min / Max	20.3 / 29.2	25.1 / 34.4	

	Med [IQR]	22.2 [21.7;23.0]	28.9 [27.7;30.0]	*p* value: <0.0001 (Wilcoxon rank sum test)
	Mean (SD)	22.5 (1.6)	29.0 (1.9)	
	n	51 (0)	72 (32)	
10wk	Min / Max	20.9 / 31.4	26.0 / 35.3	*p* value: <0.0001 (Wilcoxon rank sum test)
	Med [IQR]	23.3 [22.5;24.0]	30.1 [29.0;31.2]	
	Mean (SD)	23.4 (1.7)	30.1 (2.0)	
	n	51 (0)	72 (32)	
11wk	Min / Max	21.1 / 32.4	26.6 / 36.1	*p* value: <0.0001 (Wilcoxon rank sum test)
	Med [IQR]	23.6 [23.1;24.6]	31.0 [29.9;32.0]	
	Mean (SD)	24.1 (2.1)	31.0 (2.0)	
	n	51 (0)	72 (32)	
12wk	Min / Max	21.4 / 32.8	27.9 / 37.2	*p* value: <0.0001 (Wilcoxon rank sum test)
	Med [IQR]	24.3 [23.6;24.8]	32.0 [30.3;32.9]	
	Mean (SD)	24.4 (1.9)	31.7 (2.1)	
	n	51 (0)	72 (32)	
13wk	Min / Max	22.6 / 34.5	27.7 / 37.5	*p* value: <0.0001 (Wilcoxon rank sum test)
	Med [IQR]	24.9 [24.1;25.8]	32.5 [30.8;33.7]	
	Mean (SD)	25.4 (2.2)	32.3 (2.1)	
	n	50 (1)	72 (32)	
P100	Min / Max	23.3 / 34.2	28.5 / 38.4	*p* value: <0.0001 (Wilcoxon rank sum test)
	Med [IQR]	25.0 [24.4;25.9]	33.0 [31.4;34.4]	
	Mean (SD)	25.6 (2.2)	33.0 (2.3)	
	n	44 (7)	62 (42)	

Table S2. Number of male and female offspring per exposure group. Numbers are declining due to allocation of animals to different experiments and euthanasia for organ harvest.

	**Males (*n*)**				**Females (*n*)**			
	Vehicle	BPA	BP-3	BPA+BP-3	Vehicle	BPA	BP-3	BPA+BP-3
**P1**	31	37	50	48	52	59	65	55
**P7**	35	34	56	51	56	59	66	54
**P14**	34	34	50	48	57	60	66	54
**P21**	34	26	38	38	57	52	51	48
**wk4**	23	27	32	24	42	48	42	44
**wk5**	23	27	32	24	10	15	12	14
**wk6**	23	27	32	24	10	15	12	14
**wk7**	23	27	32	24	10	15	12	14
**wk8**	23	27	32	24	10	15	12	14
**wk9**	15	18	24	15	10	15	12	14
**wk10**	15	18	24	15	10	15	12	14
**wk11**	15	18	24	15	10	15	12	14
**wk12**	15	18	24	15	10	15	12	14
**wk13**	15	18	24	15	9	15	12	14
**P100**	11	14	24	13	6	14	12	12
**Early growth**	23	25	32	24	10	15	12	14
**Late growth**	10	14	24	13	6	15	11	12
**Mean growth**	17	18	24	15	10	15	11	13

Table S3. Statistical results of Two-way ANOVA for body mass analysis of EDC exposed offspring from P1 to P100.

Two-way ANOVA	Ordinary				
			**Male offspring**		
**Alpha**	0,05				
**Source of Variation**	% of total variation	*p* value	*p* value summary	Significant?	
**Interaction**	0.07857	0.0003	***	Yes	
**Age**	95.10	<0.0001	****	Yes	
**Treatment**	0.04667	<0.0001	****	Yes	
**ANOVA table**	SS (Type III)	DF	MS	F (DFn, DFd)	*p* value
**Interaction**	165.2	42	3.932	F (42, 1536) = 1,963	0.0003
**Age**	199922	14	14280	F (14, 1536) = 7128	<0.0001
**Treatment**	98.10	3	32.70	F (3, 1536) = 16,32	<0.0001
**Residual**	3077	1536	2.003		
**Data summary**					
**Number of columns (Treatment)**	4				
**Number of rows (Age)**	15				
**Number of values**	1596				
			**Female offspring**		
**Alpha**	0,05				
**Source of Variation**	% of total variation	*p* value	*p* value summary	Significant?	
**Interaction**	0.03023	0.9323	ns	No	
**Age**	95.78	<0.0001	****	Yes	
**Treatment**	0.03378	<0.0001	****	Yes	
**ANOVA table**	SS (Type III)	DF	MS	F (DFn, DFd)	*p* value
**Interaction**	33.35	42	0,7941	F (42, 1529) = 0.6933	0.9323
**Age**	105654	14	7547	F (14, 1529) = 6589	<0.0001
**Treatment**	37.27	3	12.42	F (3, 1529) = 10.85	<0.0001
**Residual**	1751	1529	1.145		
**Data summary**					
**Number of columns (Treatment)**	4				
**Number of rows (Age)**	15				
**Number of values**	1589				

Table S4. Number of adipocytes (*n*) included in each decile (D1-D10) and their respective minimal (Min) and maximal (Max) area. All values were calculated based on the entire population of adipocytes of male and female control (vehicle) groups.

	**Males (Vehicle)**			**Females (Vehicle)**		
	Min [μm^2]	Max [μm^2]	*n*	Min [μm^2]	Max [μm^2]	*n*
**D1**	51.29	246.31	122	50.15	151.15	226
**D2**	246.74	484.69	121	151.79	278.80	225
**D3**	492.31	756.82	122	279.08	400.21	226
**D4**	760.74	1003.64	121	400.35	540.60	225
**D5**	1011.01	1222.96	122	540.78	669.35	226
**D6**	1229.19	1458.41	121	669.78	810.28	225
**D7**	1459.80	1740.45	121	811.60	1004.10	224
**D8**	1750.10	2119.00	122	1005.06	1235.99	225
**D9**	2121.60	2658.25	121	1236.63	1592.07	224
**D10**	2661.85	5880.90	122	1592.14	4148.71	225
total			1215			2251

Table S5. Statistical results of Two-way ANOVA for adipocyte area distribution across deciles.

**Two-way ANOVA**	**Ordinary**				
		**Male offspring**			
**Alpha**	0,05				
**Source of Variation**	% of total variation	*p* value	*p* value summary	Significant?	
**Interaction**	18.66	<0.0001	****	Yes	
**Percentile**	16.45	<0.0001	****	Yes	
**Treatment**	4.608e-016	>0.9999	ns	No	
**ANOVA table**	SS (Type III)	DF	MS	F (DFn, DFd)	*p* value
**Interaction**	1104	27	40.88	F (27, 290) = 3.169	<0.0001
**Percentile**	973.2	9	108.1	F (9, 290) = 8.382	<0.0001
**Treatment**	2.726e-014	3	9.087e-015	F (3, 290) = 7.044e-016	>0.9999
**Residual**	3741	290	12.90		
**Data summary**					
**Number of columns (Treatment)**	4				
**Number of rows (Percentile)**	10				
**Number of values**	330				
		**Female offspring**			
**Alpha**	0,05				
**Source of Variation**	% of total variation	*p* value	*p* value summary	Significant?	
**Interaction**	3.190	0.9993	ns	No	
**Percentile**	6.565	0.0295	*	Yes	
**Treatment**	8.790e-017	>0.9999	ns	No	
**ANOVA table**	SS (Type III)	DF	MS	F (DFn, DFd)	*p* value
**Interaction**	290.2	27	10.75	F (27, 260) = 0.3409	0.9993
**Age**	597.1	9	66.35	F (9, 260) = 2.104	0.0295
**Percentile**	7.995e-015	3	2.665e-015	F (3, 260) = 8.453e-017	>0.9999
**Residual**	8197	260	31.53		
**Data summary**					
**Number of columns (Treatment)**	4				
**Number of rows (Percentile)**	10				
**Number of values**	300				
